# Low-Intensity Pulsed Ultrasound Modulates RhoA/ROCK Signaling of Rat Mandibular Bone Marrow Mesenchymal Stem Cells to Rescue Their Damaged Cytoskeletal Organization and Cell Biological Function Induced by Radiation

**DOI:** 10.1155/2020/8863577

**Published:** 2020-09-01

**Authors:** Rong Zhang, Zhaoling Wang, Guoxiong Zhu, Gaoyi Wu, Qingyuan Guo, Hongchen Liu, Lei Chen

**Affiliations:** ^1^Institute of Stomatology & Oral Maxilla Facial Key Laboratory, Chinese PLA General Hospital, No. 28 Fuxing Road, Beijing 100853, China; ^2^The Second Department of Naval Recuperation, First District of Recuperation, Armed Police Yantai Special Service Center, No. 191 Huanshan Road, Zhifu District, Yantai, Shandong Province 264000, China; ^3^State Key Laboratory of Military Stomatology, School of Stomatology, The Fourth Military Medical University, Xi'an, Shaanxi Province 710032, China; ^4^Department of Orthodontics, School and Hospital of Stomatology, Cheeloo College of Medicine, Shandong University & Shandong Key Laboratory of Oral Tissue Regeneration & Shandong Engineering Laboratory for Dental Materials and Oral Tissue Regeneration, No. 44-1 Wenhua Road West, Jinan, Shandong Province 250012, China; ^5^Department of Stomatology, No.960 Hospital of PLA, No. 25 Shifan Road, Jinan, Shandong Province 250000, China; ^6^Department of Stomatology, Qingdao Municipal Hospital Group, Qingdao University, Qingdao, Shandong Province 266011, China

## Abstract

Osteoradionecrosis of the jaw (ORNJ) is an infrequent yet potentially devastating complication of head and neck radiation therapy. Low-intensity pulsed ultrasound (LIPUS) has been widely accepted as a promising method for the successful management of ORNJ, but the mechanism remains unclear. In this study, the effects of LIPUS on cytoskeletal reorganization, cell viability, and osteogenic differentiation capacity of rat mandible-derived bone marrow mesenchymal stem cells (M-BMMSCs) induced by radiation were determined by immunofluorescence staining, CCK-8 cell proliferation assay, quantification of alkaline phosphatase (ALP) activity, alizarin red staining, and real-time RT-PCR, respectively. Moreover, the involvement of the RhoA/ROCK signaling pathway underlying this process was investigated via western blot analysis. We found that radiation induced significant damage to the cytoskeleton, cell viability, and osteogenic differentiation capacity of M-BMMSCs and downregulated their expression of RhoA, ROCK, and vinculin while increasing FAK expression. LIPUS treatment effectively rescued the disordered cytoskeleton and redistributed vinculin. Furthermore, the cell viability and osteogenic differentiation capacity were also significantly recovered. More importantly, it could reverse the aberrant expression of the key molecules induced by radiation. Inhibition of RhoA/ROCK signaling remarkably aggravated the inhibitory effect of radiation and attenuated the therapeutic effect of LIPUS. In the light of these findings, the RhoA/ROCK signaling pathway might be a promising target for modifying the therapeutic effect of LIPUS on osteoradionecrosis.

## 1. Introduction

Osteoradionecrosis of the jaw (ORNJ) is an infrequent yet potentially devastating complication of head and neck radiation therapy (RT) resulting from the nonspecificity of radiation. It may be associated with pain, infection, disability, and a diminished quality of life [1, 2]. To date, many preventive approaches of ORNJ have been proposed, including debridement surgery, antibiotic medicine, pentoxifylline and tocopherol prophylaxis, and hyperbaric oxygen therapy. However, these treatment effects are still unsatisfied [3, 4]. In our previous studies, we have proven that low-intensity pulsed ultrasound (LIPUS) could be a promising method for the successful management of ORNJ [5], but the mechanism remains unclear.

Bone marrow-derived mesenchymal stem cells (BMMSCs), an important component of bone tissue, play a pivotal role in both the formation and remodeling process of bone. The damage of BMMSCs can be identified in many kinds of bone diseases, including ORNJ. Moreover, the rescue of impaired function of BMMSCs is also beneficial to the ORNJ recovery [6]. When the cells are exposed to radiation, the cytoskeleton and morphology may change as well as the related gene expression. It is widely accepted that the biological functions of cells are closely related to the maintenance of physiological cytoskeleton arrangement. The early response to radiation involves many molecular signals, and GTPase RhoA has been shown to be related to the increased vascular permeability induced by radiation [7]. Moreover, RhoA and its effector Rho-associated kinase (ROCK) are the major signaling molecules affected by radiation, and the RhoA/ROCK signaling pathway definitely mediates numerous important cellular biological activities. It has been reported that the Rho family and ROCK are involved in cytoskeletal reorganization and the connection between cells. However, the mechanism by which radiation affects the cytoskeleton is poorly understood.

Recently, the therapeutic effect of LIPUS on the damage to bone has drawn increasing attention [8]. LIPUS has been used in the treatment of fresh and nonunion fractures. Many clinical trials in animals and patients have demonstrated that LIPUS shows good performance in fracture treatment, resulting in reduced healing time [9]. In terms of curing radiation related injuries, animal experiments have shown that LIPUS can improve the healing of damaged bone and lead to better conjunction between the implant and the bone after radiation. However, no related research has explored the mechanisms of improved osseointegration induced by LIPUS. The RhoA/ROCK signaling pathway has been reported to participate in cell responses to radiation, such as the reorganization of the cytoskeleton, adhesion, and viability [10]. Unfortunately, whether the RhoA/ROCK signaling pathway mediates cells recovery from radiation exposure induced by LIPUS remains unknown.

Therefore, the aim of the present study is to investigate the effects of LIPUS on the reorganization of the cytoskeleton and biological functions of rat M-BMMSCs after radiation exposure and to elucidate the involvement of the RhoA/ROCK signaling pathway in this process in order to reveal the potential mechanisms underlying the therapeutic effects of LIPUS.

## 2. Materials and Methods

### 2.1. Ethics Statement

Prior approval from the Animal Care and Use Committee of the School of Stomatology at Shandong University was obtained in accordance with the international guidelines for care in animal research. The protocol (Permit Number: GD201701) was approved by the Committee on the Ethics of Animal Experiments of the School of Stomatology, at Shandong University. All surgeries were performed under sodium pentobarbital anesthesia, and all efforts were made to minimize rat suffering.

### 2.2. Isolation and Culture of Rat M-BMMSCs

The primary rat M-BMMSCs were isolated from 4 to 6 weeks Wistar Rats maintained in the Laboratory Animal Center of Shandong University (Shandong, China). Briefly, the rats were immersed in 75% ethanol for 5 min after sacrifice. The mouths of the rats were disinfected by alcohol cotton balls. Then, bilateral mandibular bones with muscular tissues were dissociated with aseptic ophthalmic scissors from the lower jaw joint and sterilized with 75% ethanol for nearly 5 s. All teeth were extracted using tweezers. Muscles and the periosteum were scraped off with an aseptic scalpel. The bones were washed in PBS twice. A 5 ml syringe with DMEM (Invitrogen, Grand Island, NY) was used to wash the bone marrow cavity repeatedly, and the flushing solution was collected. After centrifugation at 1000 g for 5 min, the supernatant was discarded, and the precipitate was resuspended with a complete medium containing 20% FBS with 1% penicillin/streptomycin in culture flasks. The cells were incubated at 37°C with 5% CO_2_ in the incubator. The medium was changed every three days. The cells were detached and passaged when the density reached >80% confluence, and the cells at the third passage were used for subsequent experiments. The cell morphology was observed by an inverted phase contrast microscope (Leica, Germany), and the cell surface markers (CD31, CD45, CD73, CD90, and CD105) were analyzed by flow cytometry.

### 2.3. Osteogenic and Adipogenic Differentiation Assay

Rat M-BMMSCs at P3 were seeded in 12-well culture plates (1 × 10^5^ cells/well). For osteogenic differentiation detection, when the cells reached 60-70% confluence, the medium was changed with osteogenic induction medium consisting basal medium supplemented with 10^−4^ mM dexamethasone, 10 mM *β*-glycerophosphate, and 0.2 mM ascorbic acid, and the medium was renewed every three days. Likewise, for the analysis of adipogenic differentiation, the cells were incubated with adipogenic induction medium consisting basal medium supplemented with 10 *μ*g/ml insulin, 1 *μ*M dexamethasone, 100 *μ*g/ml (0.45 mM) 3-isobutyl-1-methylxanthine (IBMX), and 0.1 mM indomethacin. Finally, alizarin red staining was used to identify the osteogenic ability, while the oil red O staining was used to detect lipid droplet formation. After the induction of osteogenic differentiation for 21 d and adipogenic differentiation for 14 d, the cells were fixed with 4% formaldehyde for 30 min at room temperature. The cells were washed with PBS twice and stained with alizarin red S or oil red O staining solution for 30 min then observed under a light microscope. For the semiquantification of alizarin red staining, the mineral nodules were eluted by the following leaching solution: 10% glacial acetic acid and absolute ethyl alcohol with a volume ratio of 8 : 2 at room temperature for 30 min. The optical density (OD) of the solution was measured at 490 nm with a plate reader.

### 2.4. Group Division and Treatments

The cells between the second and fifth passages were first divided into three groups: control group (A), radiation group (B), and inhibitor group (C). Then, the cells were further divided into four groups: control group (D), radiation group (E), ultrasound group (F), and inhibitor group (G). The A and D groups received no treatments, and the B and E groups were irradiated by 4 Gy of X-ray delivered at a rate of 1.28 Gy/min (medical linear accelerator, Siemens, USA) for 12 h. The F group was exposed to 2 h LIPUS treatment (1 MHz, 100 mW/cm^2^, 20% duty cycle) (Italian ultrasonic therapeutic apparatus, US13, Italy) after irradiation for 12 h. The C and G groups were subjected to the ROCK inhibitor Y-27632 (10 *μ*M) 30 min before the same treatment as the B group and F group.

### 2.5. Immunofluorescence Staining

The M-BMMSCs were seeded on coverslips coated with 1% gelatin and washed with PBS twice at room temperature. Then, the cells were fixed with 4% paraformaldehyde for 15 min and washed with PBS three times, followed by permeabilization with 0.5% Triton X-100 for 20 min. After blocking with 5% bovine serum albumin (BSA) for 30 min, the specimens were incubated with 2.5% FITC-phalloidin and 2.5% TRITC-vinculin containing 1% BSA and 0.1% Triton X-100 to visualize F-actin for 2 h at room temperature and were then labeled with secondary antibodies (diluted to 1 : 100) for 1 h and a Hoechst (diluted to 1 : 10) complex nucleus for 10 min. All of the procedures were conducted in a dark room to avoid light. After mounting, fluorescence imaging was performed with an LSM 700 confocal microscope (Carl Zeiss, Oberkochen, Germany). The images were processed using CLSM and analyzed with the ImageJ software.

### 2.6. Cell Viability Assay

The cells were seeded into a 96-well plate at a concentration of 5 × 10^3^ in a 200 *μ*l culture medium. After incubation for 3 days, a CCK-8 reagent was added to every single well to a volume of 10% (20 *μ*l/well), followed by incubation for another 2 hours at 37°C, 5% CO_2_ in a humidified atmosphere. The optical density was measured at 450 nm using a microplate reader (Bio-Rad, Hercules, CA, USA).

### 2.7. Quantification of Alkaline Phosphatase (ALP) Activity

The cells (1 × 10^4^/ml) were seeded into 12-well plates and incubated for 5 days in an osteogenic differentiation medium. On day 5, the cells were removed from the culture plate using Triton X-100 (0.1%, *w*/*w*, in distilled water) and sonicated for 10 min on ice. The lysates were centrifuged for 15 min at 10,000×g, at 4°C. The supernatant was diluted and collected for an ALP activity assay and BCA protein quantification according to the manufacturer's instructions. Absorbance was read at 450 nm and 520 nm for the ALP assay and the protein assay, respectively.

### 2.8. Quantitative Real-Time RT-PCR

M-BMMSCs were cultured in osteogenic induction medium for 7 days as previously described. Total RNA was subsequently extracted using RNAiso Plus following the manufacturer's instructions (Takara, Japan). Reverse transcription was performed with PrimeScript® RT reagent Kit with gDNA Eraser (Takara, Japan). Gene expression of Runx2, ALP, and OCN was detected. Glyceraldehyde-3-phosphate dehydrogenase (GAPDH) was used as a control for normalization. Quantitative real-time RT-PCR was performed with SYBR® Premix Ex Taq™ (Takara, Japan). The conditions of the qPCR were as follows: 95°C for 30 s, 40 cycles at 95°C for 50 s and 60°C for 20 s, and 65°C for 15 s. Each sample was analyzed in triplicate. The primers used for the quantitative real-time RT-PCR are listed in Table 1.

### 2.9. Western Blot Analysis

Western blot was performed as previously described [11]. Briefly, the total cellular proteins were extracted, subjected to SDS-page electrophoresis, and transferred to PVDF membranes using standard procedures. The primary antibodies included mouse monoclonal anti-rat antibodies against RhoA, ROCK-1, vinculin (hVIN-1), and FAK (1 : 1000; Abcam, Hong Kong Ltd., HK, China) and rat monoclonal anti-*β*-actin (1 : 1000; Sigma, St. Louis, USA), which was used as an internal loading control. The bands were visualized and imaged using an eECL western blot kit (CWBiotech, Beijing, China), and densitometry was performed using ImageJ.

### 2.10. Statistical Analysis

The experimental data were presented as the mean ± SEM deviation of three independent experiments. Statistical analyses were performed using Statistical Package for the Social Sciences (SPSS20.0). The Levene test is used to verify the homogeneity of variance of data. Statistical significance was assessed by using one-way analyses of variance (ANOVA) and Tukey's multiple comparison tests. A confidence level of 95% (*p* < 0.05) was considered statistically significant.

## 3. Results

### 3.1. Observation and Characterization of Rat M-BMMSCs

**Figure 1:**
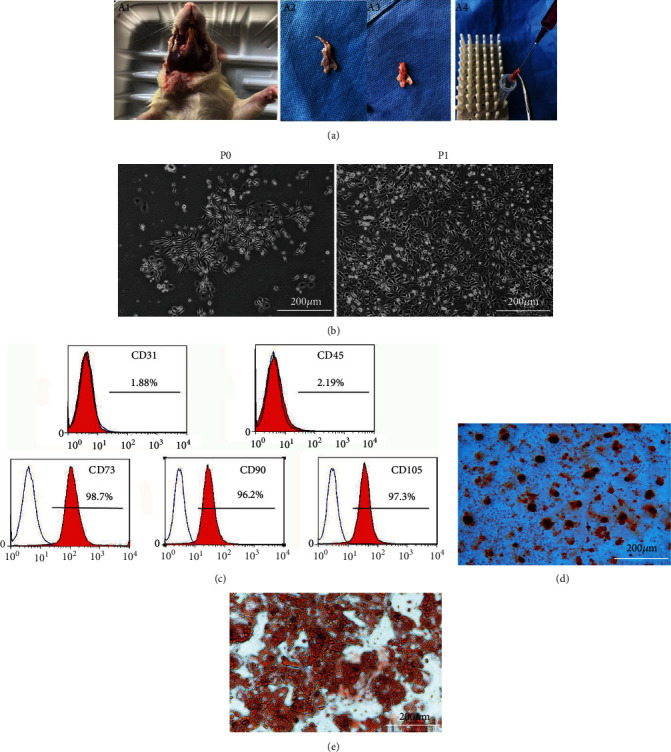
The isolation and characterization of rat mandible-derived BMMSCs (M-BMMSCs). (a) The stem cells were isolated from rat mandibular tissues. The rats were immersed in 75% ethanol for 5 min after sacrifice. (A1) The mouths were opened and the mandibles with muscles were revealed after disinfection. (A2) The intact mandibular bones were obtained after the remove of muscles. (A3) The teeth were extracted from the mandibles with tweezers in super clean bench. (A4) A 5 ml syringe with DMEM was used to wash the bone marrow cavity repeatedly, and the flushing solution was collected. (b) Observation of M-BMMSCs at P0 and P1 with the inverted phase contrast microscope (magnification, ×100). (c) Flow cytometric analysis of surface marker expression of M-BMMSCs showed negative expression of CD31 and CD45 while positive expression of CD73, CD90, and CD105 (1.88%, 2.19%, 98.7%, 96.2%, and 97.3%), respectively. (d) Alizarin red staining of M-BMMSCs after induction in osteogenic differentiation medium for 21 d. (e) Oil red O staining of M-BMMSCs after induction in adipogenic differentiation medium for 14 d. Both pictures were observed by using an inverted phase contrast microscope. Scale bars 200 *μ*m.

As it is depicted in Figure 1, the cells exhibited a fibroblast-like long spindle or polygon-shaped morphology and formed a uniform colony at P0. After subculturing, the cells at P1 spread much more, and most cells became spindle shaped with a high density. The cells negatively expressed CD31 and CD45 (1.88% and 2.19%) while positively expressed CD73, CD90, and CD105 (98.7%, 96.2%, and 97.3%, respectively) as shown in the flow cytometry analysis, which indicated that the cells were mesenchymal origin. When cultured in the osteogenic differentiation medium for 21 d and in the adipogenic differentiation medium for 14 d, obvious mineral nodules and lipid droplets could be observed by alizarin red staining and oil red O staining, respectively. These results demonstrated that the cells exhibited mesenchymal stem cell-like properties and could be used in the following experiments.

### 3.2. Radiation Damaged the Cytoskeleton and Redistributed Vinculin in M-BMMSCs

**Figure 2:**
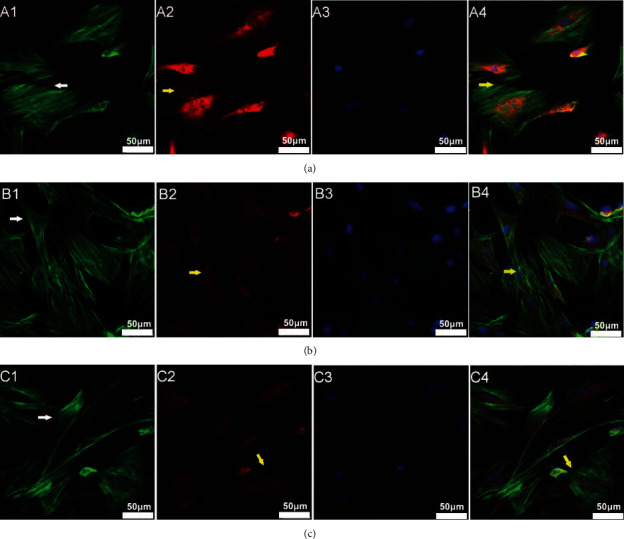
Radiation disrupted the cytoskeleton arrangement and vinculin distribution in M-BMMSCs. The cells were divided into three groups: the control group (a) that received no treatment; the radiation group (b) that was irradiated with 4 Gy of X-ray for 12 h; and the inhibitor group (c) that was subjected to Y-27632 30 min before radiation. Then, the cells were immunostained with F-actin (A1, B1, and C1) and counter stained with TRITC-vinculin (A2, B2, and C2) and DAPI (A3, B3, and C3). Graphs A4, B4, and C4 are the merged images. The white arrowheads represent the change in the cytoskeleton. The yellow arrowheads represent the change in vinculin distribution. Scale bars 50 *μ*m.

As shown in Figure 2, cells in the control group exhibited clear and intact F-actin stained in green fluorescence spanning the whole cell and well-distributed vinculin forming mature focal adhesions at the adjacent points between cells. When exposed to radiation, the whole cells turned obscure and the cytoskeleton also became randomly and sparsely located, mostly became striped distributed. In some cells, the actin became focused along the margin, or even mottled (as shown in the left part of Figure 2(b), B1). Furthermore, the vinculin decreased remarkably and aggregated surrounding the nucleus without forming obvious focal adhesions. If the RhoA/ROCK signaling pathway was blocked by Y-27632, we could see a similar manifestation of the cytoskeleton and vinculin in the C group cells.

### 3.3. Radiation Inhibited the Expression of Key Molecules in the RhoA/ROCK Signaling Pathway

**Figure 3:**
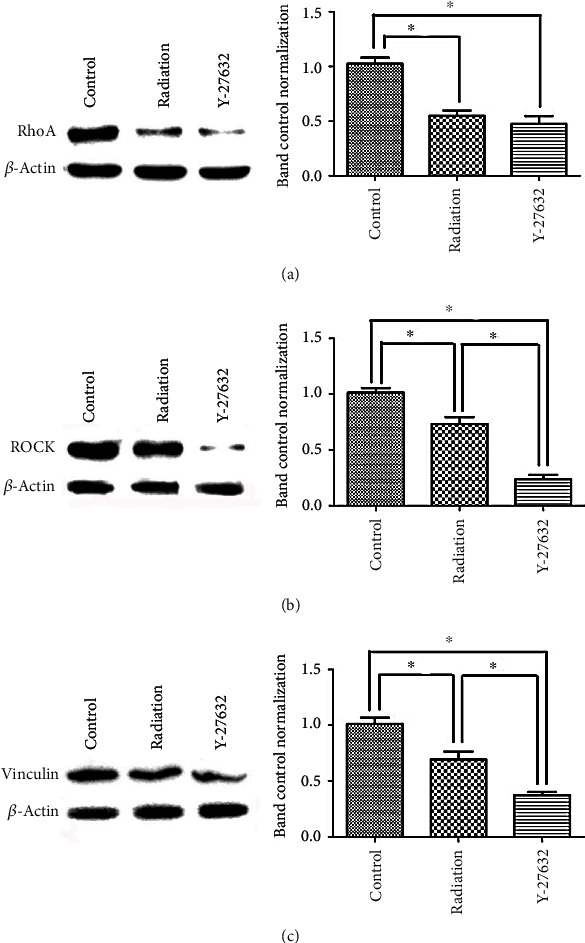
Radiation inhibited the expression of key molecules in the RhoA/ROCK signaling pathway. Cells from different groups were treated as previously described. Cell lysates were then analyzed for RhoA (a), ROCK (b), and vinculin (c) using western blot. *β*-Actin was used as an internal reference. The results are representative of those obtained in three separate experiments. Each sample was repeated in triplicate. ∗*p* < 0.05.

As demonstrated in Figure 3, the expression of RhoA, ROCK, and vinculin was lower in the radiation group than in the control group. When we blocked the RhoA/ROCK signaling pathway with Y-27632, the expression of ROCK and vinculin further decreased in the inhibitor group compared to that in the B group. However, there was no significant difference between the expression of RhoA in the radiation and inhibitor group. These results indicated that radiation may inhibit the RhoA/ROCK signaling pathway, which is critical to the expression of vinculin.

### 3.4. LIPUS Induced Cytoskeleton Remodeling and Vinculin Redistribution after Irradiation via the RhoA/ROCK Signaling Pathway

**Figure 4:**
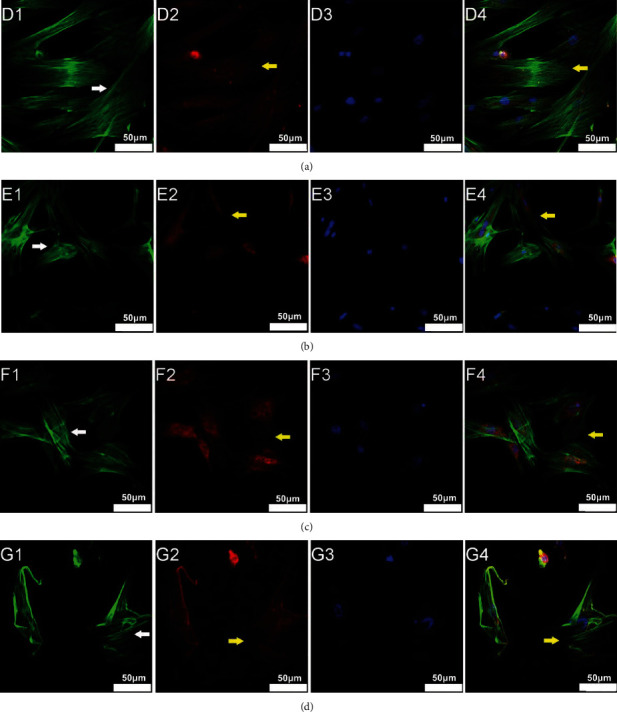
LIPUS induced cytoskeleton remodeling and vinculin redistribution after irradiation via a RhoA/ROCK signaling pathway. The cells were divided into four groups: the control group (d) that received no treatments; the radiation group (e) that was irradiated with 4 Gy of X-ray for 12 h; the LIPUS group (f) that was exposed to 2 h of LIPUS treatment after irradiation for 12 h; and the inhibitor group (g) that was subjected to Y-27632 30 min before the LIPUS treatment. Then, the cells were immunostained with F-actin (D1, E1, F1, and G1) and counter stained with TRITC-vinculin (D2, E2, F2, and G2) and DAPI (D3, E3, F3, and G3). Graphs D4, E4, F4, and G4 are the merged images. The white arrowheads represent the changes in the cytoskeleton. The yellow arrowheads represent the changes in the vinculin distribution. Scale bars 50 *μ*m.

The effects of LIPUS on the cell cytoskeleton after irradiation were demonstrated in the M-BMMSCs. As shown in Figure 4, in the control group, we could see long and orderly arranged F-actin spanning the whole cell and well-distributed vinculin forming mature focal adhesions at the adjacent points between cells. However, in the radiation group, the cell morphology became obscure, and the F-actin fibers were broken or focused along the margin of the cells, whereas the others followed a disordered mode. Vinculin conjugated only around the nucleus, while few mature focal adhesions could be found at the adjacent points between cells. When exposed to LIPUS, the cells exhibited rapid reorganization of the actin-cytoskeleton, characterized by a more thickening and expanding of stress fibers as compared with the radiation group. Meanwhile, the vinculin appeared in the cytoplasm and formed mature focal adhesions. Interestingly, when we blocked the RhoA/ROCK signaling pathway using Y-27632, the therapeutic effects of LIPUS treatment on the cytoskeleton reorganization and vinculin redistribution were obviously abrogated, confirming the critical role of an RhoA/ROCK signaling pathway in this comprehensive cytoskeletal remodeling process induced by LIPUS.

### 3.5. LIPUS Initiated the Recovery of Cell Viability and Osteogenic Differentiation Capacity after Radiation Exposure via the RhoA/ROCK Signaling Pathway

**Figure 5:**
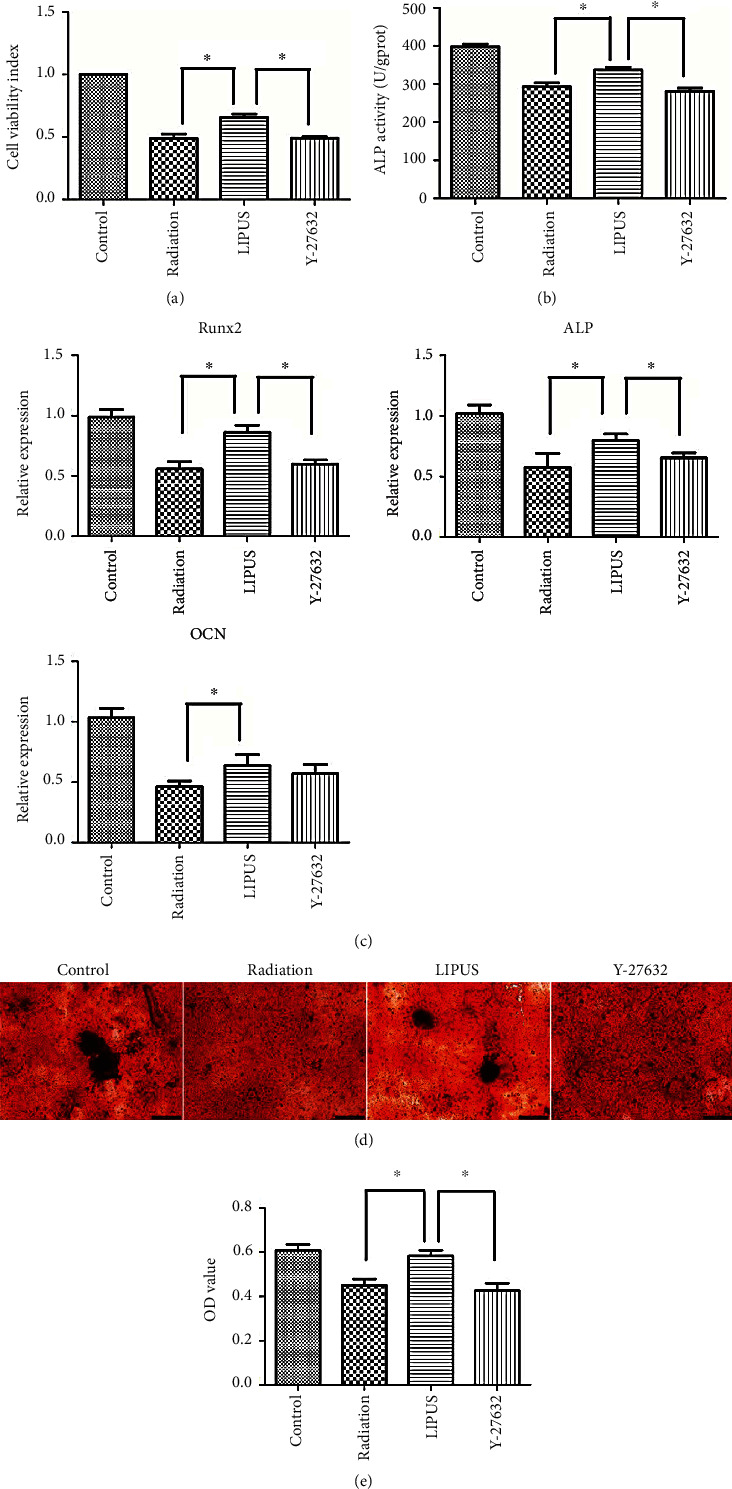
LIPUS initiated the recovery of cell viability and osteogenic differentiation capacity after radiation exposure via a RhoA/ROCK signaling pathway. In (a), CCK-8 activity at day 3 was analyzed. The optical density was measured at 450 nm using a microplate reader. In (b), group division was conducted as previously described. To assess the osteogenic differentiation activity, ALP activity was measured and scored. Absorbance was read at a wavelength of 450 nm. At the end of the experiment, the ALP levels were normalized to the total protein content. In (c), the osteogenic gene (Runx2, ALP, and OCN) expression of M-BMMSCs after incubation with osteogenic differentiation medium for 7 d was detected by quantitative real-time RT-PCR. In (d) and (e), the mineral formation of M-BMMSCs under osteogenic differentiation medium for 28 d was detected by alizarin red staining. Staining semiquantification was done by dissolving with leaching solution. All of the data represented the average ± SD from three separate experiments. Each sample was repeated in triplicate. ∗*p* < 0.05.

After observing the obvious effects of LIPUS on cytoskeleton remodeling, we next explored the influence of radiation on cell viability and osteogenic differentiation capacity and evaluated the therapeutic effect of LIPUS treatment. In our results, the cell viability was significantly lower in the radiation group than in the control group. When treated with LIPUS after radiation exposure, the cells became more energetic. The Y-27632 administration potently attenuated the therapeutic effect of LIPUS (Figure 5(a)). Alkaline phosphatase (ALP) activity is an important symbol of osteogenic capability. Here, we analyzed ALP activity using a fluorescence biotin quantitative kit 2 h after LIPUS treatment. Our results indicated that ALP activity significantly decreased in the radiation group and elevated in the LIPUS group. When the RhoA/ROCK signaling pathway was blocked by Y-27632, the recovery of ALP activity was also inhibited (Figure 5(b)). Moreover, the expressions of osteogenic genes (Runx2, ALP, and OCN) were significantly reduced when cells were exposed to radiation, which was obviously recovered by the LIPUS treatment. The application of Y-27632 remarkably attenuated the effect of LIPUS on Runx2 and ALP expression but exerted no effect on OCN expression (Figure 5(c)). These results revealed that LIPUS could induce the recovery of cell viability and osteogenic differentiation capacity after radiation via the RhoA/ROCK signaling pathway. Similarly, when subjected to radiation, the cells formed less mineral nodules as compared with the control group, which was obviously reversed by the LIPUS treatment. Y-27632 significantly impeded this effect of LIPUS by blocking RhoA/ROCK signaling.

### 3.6. LIPUS Elevated the Decreased p-FAK/FAK Ratio Induced by Radiation via a RhoA/ROCK Signaling Pathway

**Figure 6:**
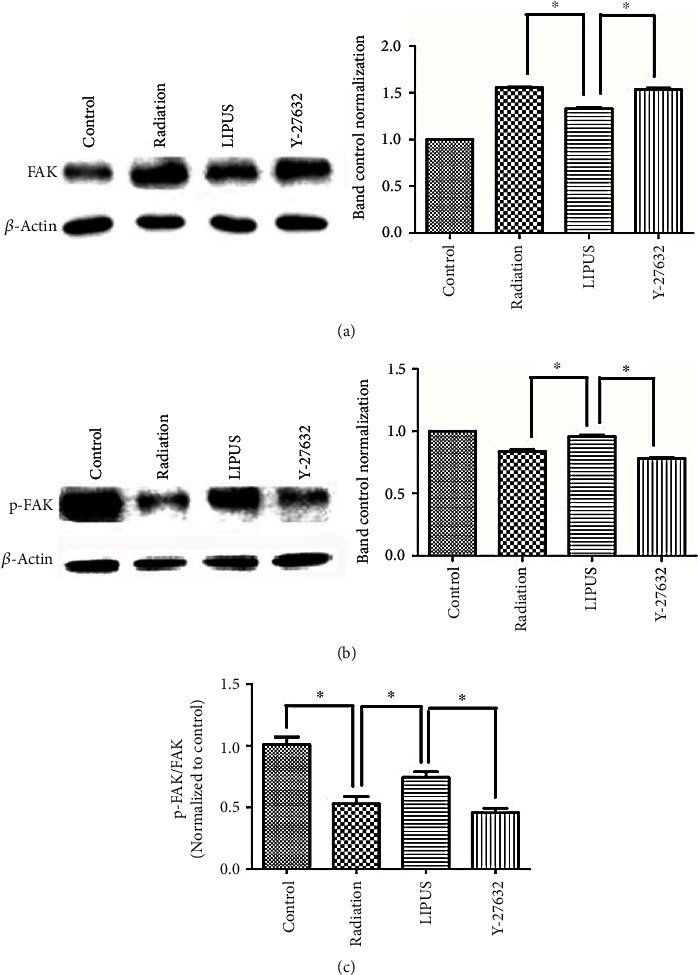
LIPUS elevated the decrease in the p-FAK/FAK ratio induced by radiation via the RhoA/ROCK signaling pathway. Cells from the different groups were treated as previously described. Cell lysates were then analyzed for FAK (a), and p-FAK (b) using western blot. *β*-Actin was used as an internal reference. The ratio of p-FAK/FAK was also determined (c). The results are representative of those obtained in three separate experiments. Each sample was repeated in triplicate. ∗*p* < 0.05.

Cell migration and adhesion are important for the regeneration of bone tissue in ORNJ. The activity of FAK is related to the formation of the focal adhesion complex, which controls the cell adhesion and migration process. Consequently, in this study, we analyzed the protein expression of p-FAK, and FAK as well as the p-FAK/FAK ratio. We found that overexpressed FAK in the E group was 1.5-fold and 1.2-fold higher than that in the D and F groups. The LIPUS treatment reduced FAK expression, which was totally abolished in Y-27632 pretreated cells (Figure 6(a)). Intriguingly, the trend of p-FAK expression in all four groups was opposite to that of FAK expression (Figure 6(b)). The ratio of p-FAK to FAK (p-FAK/FAK) obviously decreased in the E and G groups (Figure 6(c)), indicating the inhibited adhesion and migration properties of cells in these two groups. These results proved that LIPUS elevated the downregulated p-FAK/FAK ratio induced by radiation via the RhoA/ROCK signaling pathway, thereby possibly affecting the migration and adhesion of *M-BMMSCs*.

### 3.7. LIPUS Attenuated the Inhibitory Effects of Radiation on RhoA/ROCK Signaling Pathway

**Figure 7:**
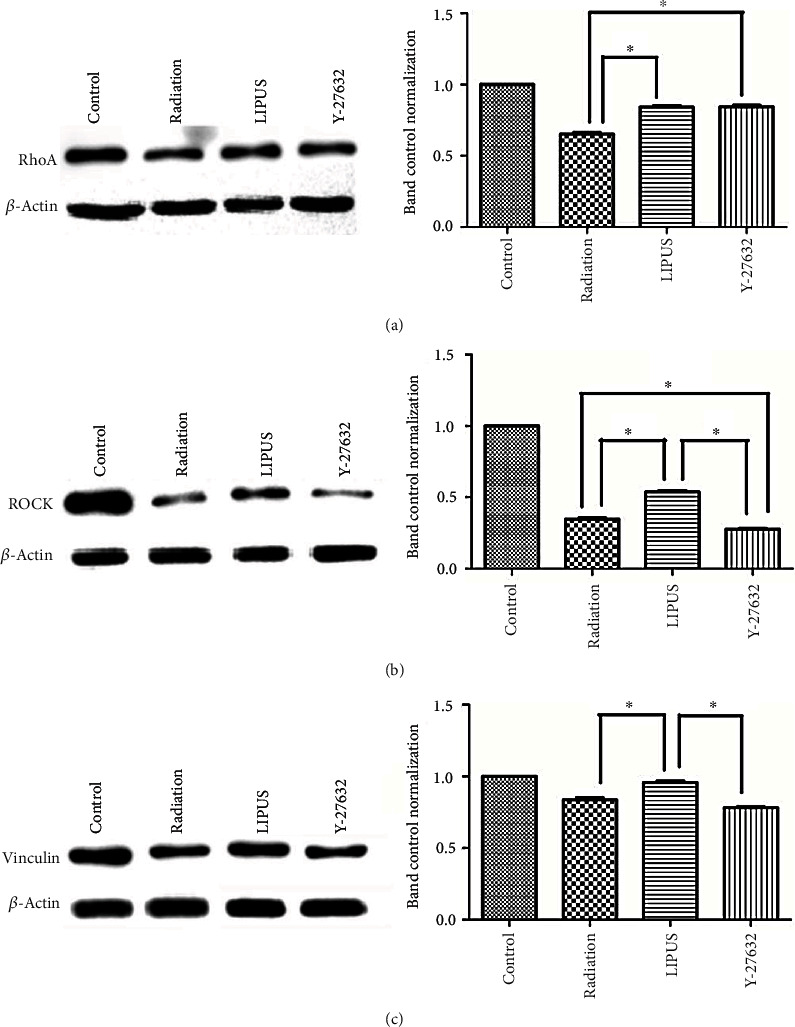
LIPUS attenuated the inhibitory effects of radiation on the RhoA/ROCK signaling pathway. Cells from the different groups were treated as previously described. The cell lysates were then analyzed for RhoA (a), ROCK (b), and vinculin (c) using western blot. *β*-Actin was used as an internal reference. The results are representative of those obtained in three separate experiments. Each sample was repeated in triplicate. ∗*p* < 0.05.

As depicted in Figure 7, radiation led to an obvious suppression of RhoA, ROCK, and vinculin in *M-BMMSCs* compared with nonirradiated cells. When the cells were administered with LIPUS, the expression of the aforementioned key molecules was obviously upregulated. Pretreatment with Y-27632 completely abolished the effect of LIPUS on the RhoA/ROCK signaling pathway, except for RhoA expression, which remained the same as that in the LIPUS treated group. These results indicate that LIPUS attenuates the inhibitory effects of radiation on the RhoA/ROCK signaling pathway.

## 4. Discussion

As an important recipient of mechanical and chemical signals in cells, the cytoskeleton is composed of a microfilament, microtubule, and intermediate filament and widely exists in eukaryotic cells. It plays an important role in maintaining cell morphology, migration, adhesion, and differentiation [12]. Furthermore, it has been proven to be closely related to phagocytosis, pinocytosis, and transmembrane signal transduction. Meanwhile, various stimuli, including radiation, mechanical force, or drugs, have been proven to cause cytoskeleton reconstruction, thus leading to abnormal changes in cell morphology [13]. In this study, we focused on the reconstruction of the cytoskeleton in M-BMMSCs after radiation and LIPUS treatment and found that cytoskeleton reconstruction exists throughout the entire process of cell damage and recovery, thereby offering a novel mechanism underlying the therapeutic effects of LIPUS on ORNJ.

Small G proteins, including Ras, Rab, Sar/Alf, and Ran subfamilies, widely participate in the regulation of cell functions [14]. Rho GTPases are important types of signal transduction molecules in eukaryotic cells and work as the molecular switch in the process. Rapid transformation between GDP and GTP efficiently transmits signals from extracellular to intracellular networks [15]. Rho GTPases may affect cell mobility through actin-myosin-regulated cytoskeleton and play a critical role in modulating actin-cytoskeleton construction [16]. The RhoA/ROCK signaling pathway can affect actin-cytoskeleton construction and play a pivotal role in cell proliferation and differentiation as well as cytoskeleton remodeling [12, 17]. Meanwhile, RhoA and its downstream effector ROCK are the key molecular signals responding to a variety of stimulus and regulating multiple cell functions, such as permeability, migration, adhesion, cell survival, and apoptosis [18, 19]. ROCK, as the downstream effector of RhoA, may cause depolymerization of stress fibers and adhesion plaque when it is inhibited [20]. In this study, we reported on the destruction of the cytoskeleton and the decrease in stress fibers in radiation-exposed M-BMMSCs, together with the significantly reduced expression of RhoA, ROCK, and vinculin, indicating the pivotal role of the RhoA/ROCK signaling pathway in radiation-induced reconstruction of the cytoskeleton. Because of the high sensitivity of vasculature to radiation, studies have focused more on the effect of radiation and LIPUS treatment on endothelial cells. Although we used different cells than previous studies, similar results arose when comparing that obtained from the endothelial cells [21]. Furthermore, our results demonstrate that the expression of key molecules in the RhoA/ROCK signaling pathway changed in correlation with cytoskeleton remodeling. Similarly, other researchers also reported the critical role of vinculin in cytoskeleton reconstruction [22]. More importantly, after blocking the RhoA/ROCK signaling pathway with Y-27632, we detected worse destruction of the cytoskeleton and less expression of key molecules in this pathway, which could not be reversed by LIPUS treatment, emphasizing the central role of the RhoA/ROCK signaling pathway in the cytoskeletal reconstruction of cells.

BMMSCs differentiation is indispensable to bone formation. ALP activity is the main differentiation parameter [23]. We indicated in this study that LIPUS treatment after radiation can promote cell viability and osteogenic differentiation. In this process, RhoA and ROCK were both activated. This biological effect was totally abrogated by the ROCK inhibitor Y-27632, suggesting that the RhoA/ROCK signaling pathway participated in LIPUS-induced recovery of cell viability and osteogenic differentiation capacity after radiation damage.

FAK, a downstream effector of ROCK, is related to the formation of a focal adhesion complex, which controls cell adhesion and migration [24]. High expression of FAK is common in cells with pathological conditions (tumors or inflammatory cells). FAK can promote the survival and development of tumor cells via various signaling pathway, including cyclin dependent kinase and cyclin independent kinase. p-FAK may combine with the downstream proteins to activate the PI3/AKT signaling pathway, thereby blocking tumor cell metastasis [25, 26]. In this study, FAK expression increased upon radiation and decreased after a cell was subjected to LIPUS, which was effectively abolished by the ROCK inhibitor Y-27632. However, the level of p-FAK expression exhibited an inverted trend. Chen et al. reported caffeine inhibited migration in glioma cells through the ROCK-FAK pathway [27]. They found that blocking ROCK with Y-27632 abrogated the caffeine-reduced P-FAK, suggesting an indispensable role of ROCK in the regulation of p-FAK, which is quite similar to our results. Here, we chose the ratio of p-FAK/FAK to illustrate the activation status of the FAK pathway, instead of using p-FAK or FAK alone. Our results indicated that the process of cell transformation from a pathological to normal status resulted from the LIPUS treatment that requires the involvement of the RhoA/ROCK signaling pathway. It has been reported that RhoA could interact with AKT, PI3K, and MARK signaling pathway to participate in the mechanotransduction process of osteoblasts [28], which warrants further investigation in our model.

## 5. Conclusions

In summary, the present study indicated that LIPUS efficiently induces the reconstruction of the damaged cytoskeleton and recovered the suppressed cell viability and osteogenic differentiation of radiation-exposed rat M-BMMSCs. The mechanism may lie in the significant elevation of the expression of RhoA, ROCK, vinculin, and the ratio of p-FAK/FAK by regulating the RhoA/ROCK signaling pathway, thereby providing experimental evidence and modifying the target of LIPUS in osteoradionecrosis treatment practice.

## Figures and Tables

**Table 1:** Primers used in quantitative real-time RT-PCR.

Target gene	Sequence (5′→3′)
ALP	Forward: AGGCTACGACACCGTCACTCTG
	Reverse: CTCCTCCTGCTTGAAGTCCTCCTTA

Runx2	Forward: TTCCAGACCAGCAGCACTCCAT
	Reverse: TTCCATCAGCGTCAACACCATCATT

OCN	Forward: CGGACCACATTGGCTTCCAG
	Reverse: GCTGTGCCGTCCATACTTTCG

GADPH	Forward: GGCAAGTTCAACGGCACAG
	Reverse: CGCCAGTAGACTCCACGACA

## Data Availability

The data used in this study are available from the corresponding author upon request.
